# “Praying in or preying on my skin”: a narrative study of transgender and gender non-conforming individuals’ experiences with religion and gender identity in India

**DOI:** 10.3389/fsoc.2025.1642404

**Published:** 2025-07-25

**Authors:** Sidhanth Ashok, T. S. Saranya

**Affiliations:** ^1^Research Scholar, School of Liberal Studies, CMR University, Bengaluru, India; ^2^Department of Clinical Psychology, School of Liberal Studies, CMR University, Bengaluru, India

**Keywords:** religion, gender non-conforming, LGBTQIA+ and faith, narrative analysis, conversion therapy

## Abstract

This narrative analysis paper explores how gender non-conforming individuals interact with faith across Indian religious traditions. Drawing upon four in-depth semi-structured interviews with the participants: Participant A, a trans Muslim man; Participant B, a transgender Hindu woman; Participant C, a gender-fluid Catholic; and Participant D, a non-binary Protestant, this study employs an inductive narrative analysis to help trace how queerness and spirituality are shaped by the structures of religious indoctrination, gendered discipline, and theological exclusion. The participants recount experiences of conversion therapy, spiritual correction, and ostracization from ritual space, with many describing an internalized fear of God’s punishment. Most poignantly, the themes of “God’s silence,” “Spiritual exile,” and “Reclamation of Sacred Identity” take shape from the narratives and speak to the theological and emotional labor of reconciling the expression of gender identity and shaping faith. All four subjects show significant personal spiritual agency, despite institutional failures, which they functionally demonstrate through their own reinterpretations of scriptures, personal ritual practices, and organized queer faith communities. These narratives also push back against the gender binaries associated with a narrow set of constructs in Indian religious discourse, and offer a counter-theology that’s based on embodiment, defiance, and reinterpretation. Additionally, this study shows the ongoing need to construct religious belonging beyond cisnormative practices, and places queer spirituality as a reality, instead of reality-anomaly.

## 1 Introduction

Religion plays a major role in the sociocultural life of India because it is part of what shapes identity, community belonging, and gender specification. For gender non-conforming individuals (transgender, non-binary, gender-fluid persons), religion acts as a threshold space that lies between marginalization and reclaiming a spiritual self (Bashid and Prabha, 2023; Kapur, 2024). Although queer identities gained legal recognition after Section 377 was read down in 2018, many religious and cultural scripts continue to discipline queerness through theological exclusion and ritual exclusion. This paper seeks to examine how four queer individuals navigate, and actively reconstruct their spiritual identities through inductive narrative analysis from Islamic, Hindu, and Christian (Catholic and Protestant) traditions. Historical and scriptural anecdotes are often richer than what contemporary orthodoxy will admit with respect to gender pluralism, Tritiya prakriti, that is third gender, and deities such as Ardhanarishvara (Alisha, 2018). As complicated evidence, Saria (2021) reported that hijras, despite being deemed part of out-caste, still perform ritual roles as part of childbirth, marriage, or divine mediation, which demonstrates their spiritual permanence. Nevertheless, institutional religion in post-colonial India has largely conformed to cisnormative and patriarchal expectations. Hindu orthodoxy has been reconstructed through hierarchical colonial-nationalist lenses. As a result, queer and feminist theologies have been suppressed (Graham and Sundarraman, 2018). Likewise, mainstream interpretations of Christianity and Islam in India frame queer identities as morally, spiritually deviant and immoral (Jones, 2021; Kapur, 2024).

Yet in this exclusion, queer people display what is referred to as ‘illicit spiritual personhood’; ‘not only making forms of worship, re-interpreting scripture, and performing rituals that defy institutional imperatives; but also creating new forms of sacred belonging’. As an example, an ethnographic examination of Yellamma’s devotee community demonstrates the gender-transgressive roles assumed as divine callings in a matrifocal spiritual order and that male-sexed bodies should serve as priests, serving goddess Yellamma. Similarly, retelling of mythological narratives by Devdutt Pattanaik has unveiled suppressed queer figures in Hindu myths, many of which are difficult for hegemonic theologies to accept (Alisha, 2018).

Nevertheless, exclusion oftentimes leads to resistance. Ramberg (2024) refers to an “illicit spiritual personhood,” which is an expression of a non-normative faith that engages with a different reading of scripture and builds new sacred spaces. Similarly, the retellings of Devdutt Pattanaik highlight marginalized queer figures in Hindu myth (Alisha, 2018). The current study builds on these interventions to document how participants A, B, C and D have created counter-theologies based on embodiment, resistance, and reclamation. In ritual practice, re-interpretation, and collective belonging, they may have transformed queer spirituality into a legitimate and generative form of religious practice in India. As Kasmani (2024) suggests, “queering religion” is not only a matter of inclusion, but one of re-orienting religious epistemologies toward ambiguity, multiplicity and non-normative belonging.

The research is grounded in a theoretical framework that synthesizes intersectionality, queer theology, and postcolonial feminist theory. Intersectionality, as developed by Crenshaw (1991), focuses on the intersection of various marginalities like gender identity, caste, class, and religion. Queer theology (Yip, 2005; Wilcox, 2003) provides a prism to re-read religious texts by affirming, non-normative theological visions. Postcolonial feminist theory (Mohanty, 1988) challenges colonial legacies within religious institutions and their gendered power dynamics. The approach is used to analyze further themes of ‘spiritual exile’ and ‘reclamation of sacred identity.

## 2 Methodology

### 2.1 Research design

This study employed a qualitative narrative analysis within an interpretivist framework to explore how gender non-conforming individuals engage with religious belief and practice across Indian spiritual traditions. Narrative inquiry enables a contextualized, emic understanding of identity, meaning-making, and socio-theological negotiation (Riessman, 2008). Rather than focusing solely on what is told, narrative analysis attends to how coherence is created in response to spiritual trauma, exclusion, and reclamation, which aligns with the study’s focus on themes like spiritual exile, God’s Silence, and Reclamation of Sacred Identity.

### 2.2 Participant recruitment and selection

To recruit participants, purposive sampling was employed. Participants were recruited through LGBTQ+ spirituality networks and community social media, and the specific inclusion criteria were that the participants (1) identified outside of the cisnormative binary, and (2) they currently or previously housed a religious practice that came from India. The sample consisted of: Participant A, a trans Muslim man, Participant B, a transgender Hindu woman, Participant C, a gender-fluid Catholic, Participant D, a non-binary Protestant (Table 1).

**Table 1:** Participant demographics.

Participant	Age	Gender identity	Assigned sex at birth	Religion
A	24	Trans man	Female	Islam
B	29	Trans woman	Male	Hinduism
C	24	Gender fluid person	Female	Catholic Christianity
D	27	Non-binary person	Male	Protestant Christianity

### 2.3 Ethical considerations

#### 2.3.1 Study protocol approval

The study protocol was approved by the School of Liberal Studies ethical committee CMR University, Bangalore after proper scrutiny by the ethical committee members.

#### 2.3.2 Informed consent

Prior to interviewing the participants, signed informed consent was collected in order to consent to participate in the research and were aware they could withdraw from the research at any time without any penalty.

Written informed consent was also obtained from the individuals for the publication of any potentially identifiable data, including direct quotations, demographic details (such as age, gender identity, and religious affiliation), and personal experiences included in this article.

#### 2.3.3 Participant identities

All the identifiable information, regarding the participants identities were removed from the manuscript.

#### 2.3.4 Participant wellbeing and reflexive practice

Considering the potential sensitivity of talking about religious trauma and gender identity, Trauma-informed interviewing practices were relied upon. At the end of the interviews, participants were provided with optional mental health resources, which complied with ethical requirements for working with vulnerable groups (Liamputtong, 2007). Throughout the interview process, reflexive ethical practice was exercised as well as mindfulness of power dynamics with the participants (Finlay, 2002).

### 2.4 Data collection

Data for this study was collected using semi-structured in-depth interviews lasting 60–90 min. This approach resulted in the depth and flexibility of the interviews while yielding reasonably consistent narratives. The participants were encouraged to share reflective stories of their experiences of discovering their gender in a religious context through open-ended prompts that asked questions like, “Can you describe your earliest memory of being aware of your gender in a religious context?” Other narrative-eliciting questions investigated the participants’ religiously affirming/exclusionary teachings, and how participants have negotiated historically-held interpretations of their faith with their gender identity (Riessman, 2008). Participants determined their interview locations (in-person at neutral sites, virtual via secure video-conferencing), with the main consideration being their comfort and privacy. Participants were also encouraged to bring sacred objects/texts to assist in remembering and embodied reflection. Interviews were audio recorded (with consent) and transcribed verbatim and included non-verbal expressions and affective cues. Transcripts were returned to participants for member checking, which established credibility and enabled participants to make editorial amendments (Lincoln and Guba, 1985). With permission, and whenever necessary, translations and back-translations were made to ensure semantic meaning and cultural significance. All data is de-identified, securely stored and solely used for this research.

### 2.5 Data analysis

This study used narrative analysis and thematic analysis in combination to investigate both participants’ form and meaning of compilation. Narrative analysis, borrowing from Riessman (2008), acknowledges each participant’s sequence of experience, emotional position, and metaphorical language. Narrative analysis facilitated investigation of transitions, such as from commitment into disillusionment, and how participants navigated sacred reclaims and theological exile. Then, Braun and Clarke’s (2006) six-stage thematic analysis was used. Having read the transcripts several times, initial codes were generated with regard to both semantic and latent meanings (Braun and Clarke, 2006). *In vivo* codes were created from words of the participants. Codes were put together in respective themes like “God’s silence,” “Spiritual exile,” and “Reclamation of Sacred identity.” In addition, sub-themes like “rejection by religious community” and “forced dis-identification from sacred roles” arose in an iterative manner. Excerpts from the narrative in the interview were chosen for their symbolic and emotional significance. Trustworthiness was ensured by maintaining reflexive journaling (Finlay, 2002), member checking transcripts, and ethical issues (Lincoln and Guba, 1985). Manual coding approaches with the use of color-coded highlighters, thematic maps, and margin notes were utilized to immerse themselves in the data and to follow theological and emotional threads within the narratives.

While narrative analysis was employed to explore the personal coherence and emotional arc of each participant’s narrative, thematic analysis enabled cross-case synthesis of common patterns. Narrative analysis was concerned with the ‘how’ of storytelling (structure, tone, and metaphor), while thematic analysis picked up on repeated latent and semantic themes like ‘God’s Silence’ or ‘Spiritual Exile’.

a) Coding process expanded.

Line-by-line coding was initially performed separately by both authors.

Coding was both deductive and inductive, starting with *in vivo* codes.

Themes were iteratively refined through peer debriefing.

Data saturation was considered to be complete when no new codes were found in the last transcript.

b) Reflexivity and positionality.

Both researchers recognize as cisgender queer-affirming researchers committed to feminist, intersectional research practices. The first author self-identifies as a queer ally and has existing experience in TGNC support groups. Reflexivity was achieved through journaling and audit trails to try and reduce interpretive bias.

View Appendix A for a visual representation of the coding structure.

## 3 Narrative themes and analysis

### 3.1 God’s silence

The theme of God’s silence illustrates the emotional and spiritual turmoil participants experienced when they had been praying for a sense of change or understanding and heard silence. Essentially, this theme reflects feelings of devotion that participants felt when coming to terms with being unheard, invisible, and abandoned by God or some other divine creator. This theme of faith crisis, disbelief, and desire reflects the dissonance participants experienced when they sought recognition from a benevolent divine being while attempting to affirm their own gender and sexual identity.

#### 3.1.1 Unanswered prayers for change

This sub-theme highlights the participants’ early attempts to conform to religious expectations of praying to change their gender or sexual identity. Even with strong dedication to the practice and countless petitions made, the religious hierarchy and prayer yielded no results, which brought trauma and stress on their feel of relationship with the divine. The silence faced only served to reinforce the inadequacies and confusion regarding their spirituality.

*“I remember praying to Allah, begging him not to look at me as a bad Muslim because I had no control over my desire to be a boy. I cried every night, I was raised to be a good Muslim woman, and yet, whenever I had to isolate from prayers when menstruating, I would feel dysphoric and feel more pain because of that than the cramps. I would beg Allah to change it, to make me right as a boy, but he never responded. I would wonder whenever I prayed, if I was forgotten or forsaken by him.”* - Participant A (24), Muslim trans-man (Assigned female at birth).

“*Gender dysphoria has been a part of me for as far as I can remember, just as in the way my faith in God has been. I remember when I started feeling it the most, it was when I turned 16. I felt like a sinner because I questioned my identity, and the guilt got worse every time I visited the the church for prayer. I remember wanting to tell my ma about it, but I was scared because she used to judge the trans-women from the hijra community by saying that they went against the law of god’s natural creation. I used to pray to God for him to fix me, and not question my womanhood, only to wake up the next day and feel the same way.*” *-* Participant D (27), Protestant Christian, Non-binary person (They/Them, Assigned male at birth).

#### 3.1.2 Feeling abandoned by god

This sub-theme embodies the resulting spiritual rejection participants experienced due to unanswered prayers. Silence, in this case divine silence, was internalized by participants as abandonment, and they construed themselves as abandoned, unworthy, or excluded from God’s grace, which further aggravated their emotional isolation or distance, weakened their faith and ultimately exacerbated their spiritual distress.

*“I started to doubt my faith, maybe Krishna-swamy did not consider me worthy of his help because I went against how my body was created. When my Amma found out my secret through a friend who sent her photos from my private Instagram, she called me a curse, and that no god can even save me. It hit me different, because I have been a devotee of Krishna swamy ever since I was a child, and to hear that my biggest source of hope, my main guiding source, would not even accept me, crushed me and had me feel like I was beyond being saved*.” - Participant B (29), Hindu Trans-woman (Assigned male at birth).

*“I remember this one time during the month of Ramadan. I woke up at Fajr and cried during namaz. I thought if I prayed hard enough, showed how devoted I am as a good Muslim, that Allah would take these parts of me that I resented, like these breasts on my chest that carry the weight of my hatred and pain, and the voice I could not bear. But nothing happened. Days passed, years passed. And one day, I just sat there on the prayer mat thinking. maybe Allah does not want to hear me. Maybe I’m already too far gone. I am haram because I am going against Allah’s law.”* - Participant A (24), Muslim trans-man (Assigned female at birth).

*“My mother used to read out the story of Adam and Eve to my elder brother and me before we went to bed, when we were both kids. I mean, yeah, when you are raised in a catholic christian household, you are most likely raised to be deeply rooted within your belief in Jesus. The point I am making is that I always felt a pang of guilt because even as a kid, it never felt quite right, but that story of Adam and Eve was a constant reminder that there are only two genders, and here I was, having confusion on what I was, guess god did not build me as perfect as my family claimed he did”* - Participant C (24), Catholic Gender-fluid person (Assigned Female at birth).

### 3.2 Spiritual exile

The theme of spiritual exile captures the experience of participants feeling a sense of exile from their religious community and rituals after being authentic to their gender and sexual identity. This theme covers participants being explicitly rejected from religious organizations by religious institutions, but also the more implicit experience of dislocation from religious spaces and religious roles linked to gender. This theme demonstrates participants being made to feel like an outsider in religious spaces that were once sacred as a means to provide most participants with purpose, meaning, and connection; instead, participants were left with feelings of impotent isolation and disenchantment.

#### 3.2.1 Rejection by religious community

This sub-theme captures how participants experienced marginalization, judgment, and exclusion from their religious communities after revealing or expressing their gender or sexual identities. This exclusion shows up in various ways: from subtle alienation and gossip to losing community positions and access to rituals. For some participants, it included having to go through extreme hardships such as conversion therapy, which is usually packaged as spiritual healing or correction, and is peddled to he family or relatives of the victims that the idea that their identities were unfaithful. Participants experienced deep feelings of betrayal, spiritual displacement, and disconnection from the very institutions that once reinforced their belief in divine love and belonging.

*“Mhm, yeah, running away from home was the best decision I ever made. I remember the literal hell I suffered, and all I feel is a sense of anger and resentment. I was 18 when my parents started obsessing about marriage, My father kept talking about getting me married off early to avoid zina. I kept pushing it away as hard as I could, but when I turned 22, they forcefully tried to get me married, and that’s when I screamed that I believed I am a boy, and that I am only attracted to women, not men. Within the next four hours, they took me to a maulana in the city. The maulana said that my soul was being held hostage by a shayatin, which is an evil spirit in Islam, that was making me break Allah’s law. I was fed hideous concoctions, and prayers were chanted to get the evil shayatin out of me, surprise, it did not.” -* Participant A (24), Muslim trans-man (Assigned female at birth).

*“Eventually, I told my mother about my identity as a gender fluid person, she took it horribly, she and my father took me to a Christian counsellor. The counsellor seemed really sweet; she was soft-spoken and seemed like she was genuinely trying to understand me. Boy was I wrong, she later told my parents that the confusion in identity is because of how modern day media is brainwashing the youth into sin, or whatever that meant. That was the first time I ever opened up about my struggles with sticking to just a single gender identity in great detail. I was forced to practice a regimented routine of prayer sessions to supposedly cleanse my soul, and forcefully lie and acknowledge that my thoughts are impure and devoid of God’s grace. I only managed to make it stop by admitting that I was wrong. I did this for my mental peace because if not, I do not think I would have survived.”* - Participant C (24), Catholic Gender-fluid person (Assigned Female at birth).

#### 3.2.2 Forced disidentification from sacred roles

This sub-theme investigates how all participants were forced to relinquish or repress their participation in religious roles, leadership roles, or rituals since they disclosed their gender or sexual identities. Their authentic selves were not consistent with traditional expectations, and they were left with no choice, to either cover up their true identity or step away fully. This loss stripped them of both spiritual purpose and belonging, and subjected them to an even deeper sense of exile, as they removed themselves from that which gave life to their faith.

*“In my family, the eldest son performs the rituals, since we are Brahmins, one of them is lighting the deepam, to lead the annual Shraddham for our ancestors. That was my role. I grew up memorizing and śloka chanting with my father during Amavasya. But the year I started wearing sarees, they said I had become impure. My uncle whispered, ‘A hijra cannot lead the rites of a Brahmin.’ During my grandfather’s death anniversary, they made my younger cousin light the lamp while I stood at the door, like I was some ghost. I wasn’t even allowed to enter the pooja room. I felt like I’d been erased. As if all those years I served the divine meant nothing the moment I claimed my truth.”* - Participant B(29), Hindu Trans-woman (Assigned male at birth).

*“Before openly coming out of the closet as non-binary, every Christmas morning, Appa would ask me to say grace before breakfast. It was our thing, just the two of us, palms joined, while Amma served dosa and plum cake. But after I told them I’m non-binary, that ritual vanished like it never existed. That year, Appa just said grace himself without even looking at me. It was like I’d been spiritually erased in my own home. Later, I heard Amma whisper to my aunt, ‘How can someone who rejects God’s design bless the food?’ That sentence stayed with me. It was the first time I realized my faith was no longer theirs to accept*.” - Participant D (27), Protestant Christian, Non-binary person (They/Them, Assigned male at birth).

### 3.3 Reclamation of sacred identity

This main theme represents how participants reclaimed spiritual agency regardless of their gender and sexual identity, which contributed to unique asserted spiritual pathways. Participants expressed that many had consciously distanced themselves from formalized religious institutions and had, instead, formed their own spiritual communities. These chosen communities opened up possibilities such as creating safe, inclusive spaces for those who wanted an opportunity to worship and express their faith and identities. Throughout this gathering up of community participants simultaneously assembled personal spiritual practices, such as developing private rituals, establishing their own and potentially new readings of text, and speaking to the divine in intimate and non-traditional ways. Much of this process reignited spirituality as a way to heal, accept, and reclaim individual power, which was free from ancestral systems that insisted on their exclusion.

*“It has been two years, I do not go to the mosque anymore, especially being around cis gender men like the maulana who tried to fix me in the name of Allah, triggers me into a panic attack. But. I still pray to Allah, just my own way. I lay down a mat in my room, face west, and talk to Allah like He’s someone who knows me beyond all this noise. No formality, just honesty. And I think for the first time, I feel like He’s listening. Like he sees me as I am. I think accepting myself has led to Allah accepting myself. I found an online group for queer Muslims too. We meet on Zoom during Ramadan. We break fast together, read verses that do not condemn us, and if possible, some of us try to meet in person. That’s the only time I’ve ever felt faith, peace, and acceptance for my gender and sexual identity in the same breath.”* - Participant A (24), Muslim trans-man (Assigned female at birth).

*“After leaving home, I stopped going to temples. But I did not leave Krishna Swamy, He’s still with me, in my mirror, my saree, my breath. I light a deepam every evening in my room, not for ritual, but for him. For me. There’s a group of queer Hindus online who do aarti together once a month. We talk about the trans figures in mythology, such as Mohini, Ardhanarishvara, which in a lot of ways soothes my existence, that I am, in fact, real, that there were deities before me, and that alone is enough to validate that I am truly living. I never heard those stories in my house, but now they feel like they are mine. Like I was never outside my faith to begin with.”* - Participant B (29), Hindu Trans-woman (Assigned male at birth).

*“I no longer go to churches, especially after being betrayed by that counsellor, but after a period of time, I realized that perhaps, my faith is more important to me at an individual level and does not have to be attached to a specific religion. Sing hymns in my room with the pronouns changed. I say ‘they’ for God. It feels right. I joined a queer an interfaith circle sometimes we read Psalms or readings from other religious books, sometimes we just sit and breathe together. No pressure. Just people trying to hold space for something bigger than us, while still holding ourselves” -* Participant C (24), Catholic Gender-fluid person (Assigned Female at birth).

*“I still hold onto my faith in god, even if my family and I do not celebrate Christmas together, it’s fine, I will be moving out in about a year from now. I have been finding support through online protestant christian queer groups, and just knowing, even if not feeling the presence of other queer people like me who hold onto our faith in god, even though the world preaches that we do not deserve him, gives me hope”* - Participant D(27), Protestant Christian, Non-binary person (They/Them, Assigned male at birth).

## 4 Discussion

This narrative analysis study examined the spiritual journeys of four TGNC (Transgender, gender non-conforming) individuals in India and identified the horizons of spiritual struggle as beginning with an inner spiritual conflict, which progressed to community rejection, and moved on to reconstructing their identity. The first theme, “God’s Silence,” captured the emotional conflict of prayers gone unanswered in seeking transformation, and the perceived abandonment of the divine. The participants, in particular, Participant A and Participant C, expressed great personal pain associated with their theological rejection of their identity, similar to the complex spiritual struggle evident in previous studies (Exline et al., 2014). The second theme, “Spiritual Exile,” examined the intra-personal dimension of faith-based exclusion. The participants experienced the rejection of not only their religious vocation, but were also, in a sense, symbolically exiled from their community. The rejection of participation in sacred rituals, like Participant B’s temple dance or Participant D Christmas planning, exemplified their disconnection from spaces that reinforce their identities. This promotes research on the psychological costs associated with minority stress (Meyer, 2003) and the additional effects of institutional erasure. Nonetheless, all participants ultimately were involved in the Reclamation of their sacred Identity (Third theme), and were developing affirming spiritual frameworks that were greater than institutional religion. Through chosen communities, individualized rituals, and reframed theologies, they moved toward integration and healing. These findings are an example of the psychological utility of coherence of identity and spiritual agency (Rodriguez and Ouellette, 2000), as well as the transformative potential human beings possess through meaning-making (Singh et al., 2014).

The affective narratives of religious rejection in this research find resonance with Judith Butler and Trouble (1990) theory of performativity, in which the imposition of binary gender symbolizes non-conformity. Eve Sedgwick’s construction of the ‘epistemology of the closet’ (1990) accounts for the silencing that happens in religious institutions (Sedgwick, 1990). The research also supports Marcella Althaus-Reid’s ‘indecent theology’ (Althaus-Reid, 2000) by bringing to the center of attention the erotic and transgressive potential of queer spiritualities beyond institutional normality.

Sharma (In press) illustrates how religious abandonment in itself creates a narrative of resistance. Integration of tales of TGNC persons who abandon organized religion can extend ‘spiritual exile’ to more interpretive scope. Upadhyay (2020) sheds light on how caste and Hindu nationalism specifically inform queer exclusions. The manuscript now encompasses a comparative context of how postcolonial conditions condition Hinduism, Islam, and Christianity differently (Table 2).

**Table 2:** Comparative table shows the post-colonial religious impacts on queer exclusion.

Religion	Postcolonial influence	Queer exclusion mechanisms
Hinduism	Caste supremacy	Caste based gate keeping of rituals and spiritual roles
Islam	Colonial pathologization of gender variance	Moral policing and shayatin narratives
Christianity	Missionary moralism and Victorian sexual ethics	Sexual purity doctrines and conversion therapies

This comparative table underscores how colonial and nationalist heritages framed the policing of gender nonconformity in three of the world’s largest religious traditions. For Hinduism, the ideologies of caste and Hindutva have reorganized ritual status and spiritual offices through exclusionary discourse (Michaels, 2015). For Islam, colonial medicalization and moralizing discourse redescribed queerness as spiritual corruption (Nasr, 2003). For Christian missions, sexual purity regimes were often exported that pathologized gender nonconformity. These postcolonial divergences demonstrate that queer exclusion within Indian religious life is not monolithic, but rather historically contingent and organized according to intersecting ideologies of caste, purity, and nationalism.

## 5 Conclusion

To conclude, the narratives of the four study participants demonstrate the complexities and the resilience of TGNC persons in India, as they negotiate an often-fraught and tenuous religious and gender identity. These narratives revealed important findings such as (1) God’s Silence, where participants described feelings of abandonment and about prayers for change that went unanswered, and (2) Spiritual Exile, where participants found themselves rejected from their religious community and stripped of meaningful religious identities and roles. However, important as well, the narratives demonstrate a movement toward empowerment through the Reclamation of Sacred Identity, whereby the TGNC individuals rebuilt affirming communities, and redefined their spirituality positively and inclusively. Collectively, these themes illustrate that while rigid and prescribed religious norms are detrimental to the psychological and spiritual well-being of TGNC persons, all the participants’ responses are marked with remarkable resilience: creating new paths to wholeness that honor both their faith and their selves. This study offers important insights into the experience of transgender and gender non-conforming people reconciling and integrating multiple identities and advances the urgency of inclusion and understanding of TGNC people across religious contexts alluded to in the existing literature.

## 6 Implications for practice

- Mental health professionals should be aware of the psychological effects of spiritual conflict when working with clients experiencing spiritual conflict, such as feelings of shame, religious trauma, and internalized stigma (Exline et al., 2014; Singh et al., 2014). Therapy can also help clients work through grief over a lost sense of belonging to a religion, find the meanings that are important to them personally in a spiritual sense, and affirmatively integrate their identities, and resolve their conflicts regarding their spiritual identities (Rodriguez and Ouellette, 2000).
- Clergy and faith leaders should acknowledge that spiritual exclusion can be harmful and take the initiative of inclusion: for example, when certain scriptures are used to justify exclusion, re-interpret them and include TGCN individuals in ritual participation and leadership (Wilcox, 2003; Yip, 2005). Developing compassionate pastoral care and lending their voice to public advocacy will help reduce a TGNC individual’s experience of minority stress and spiritual alienation (Meyer, 2003).
- Educators and trainers should include intersectional content in psychology, theology, and social work curricula that include religion, gender identity, and sexual identity (Bilodeau and Renn, 2005). Preparing professionals to work with TGNC clients, from a religious background will help mitigate systemic barriers and increase resilience against oppression.

## 7 Limitations

The small and diverse sample (*n* = 4) limits the generalizability of this study’s findings. Each participant represented a distinct religious tradition, which limited the comparative possibilities across religious sects or denominations. All of the participants also had some connection to spirituality, excluding perspectives of those who had disengaged completely from religion. Overall, although the qualitative nature of the study captures deep individual stories, the findings are still context-specific and interpretive.

While purposive sampling guaranteed in-depth insights, the use of urban spirituality networks could have left out those living in rural, poor, or non-digital environments. This omission can be responsible for what Bauer et al. (2009) refer to as ‘informational and institutional erasure’, specifically impacting marginalized caste and class identities. Future research needs to actively include TGNC individuals belonging to SC/ST communities and rural spaces in order to record caste- and class-specific experiences of religious marginalization.

## Data Availability

The raw data supporting the conclusions of this article will be made available by the authors, without undue reservation.

## Glossary

Aarti: Aarti is a type of worship ritual in Hinduism in which light from wicks dipped in ghee or camphor is offered to one or more deities. The ritual is usually performed during puja ceremonies and represents the removal of darkness (ignorance) while inviting in light (knowledge) (Michaels, 2004). Typically, while being performed, the Aarti is circled in front of the image or idol of the deity, accompanied by devotional songs
Amavasya: Amavasya is the new moon day in the Hindu lunar calendar. Amavasya and the accompanying rituals are of religious significance. For example, several rituals are performed for Amavasya, such as fasting and memorial services for ancestors and worship for the deity. Amavasya has variations in timing from various regional calendars, where one marks the beginning of a lunar month while another calendar marks the new moon as the end of a lunar month (Chaturvedi, 2002).
Deepam: Deepam, also known as a diya, is a traditional oil lamp (light) made of clay, metal, or another material included in Hindu rituals and festivals. Lighting a deepam symbolizes the dispelling of darkness and of ignorance. Lighting a deepam invokes divine presence and blessings during worship (Stutley, 2006).
Fajr: Fajr is the first of the five daily obligatory prayers in Islam and is observed at dawn, at the point just before sunset. It is performed in two rak’ahs (units of prayer) and is considered a spiritually meaningful prayer that starts a Muslim’s worship for the day (Glassé, 2001).
Haram: In Islamic law, haram is used when discussing something that is expressly prohibited by law. Muslims consider acts that are haram sinful, while the act of abstaining from haram acts are deemed to offer rewards. Harām refers both to one’s moral conduct and legal prohibition on one’s actions (Al-Qaradawi, 1994).
Hijra: A hijra is a recognized and distinct third gender in South Asian cultures that can include individuals who are transgender, intersex, or eunuchs. Historically, hijras have provided specific social and religious roles, but are often excluded from societal norms and constructs. Legal recognition varies by country in the region (Nanda, 1999). However, the meaning of hijra is also, in South Asian contexts, used in a discriminatory manner. The term is used to insult someone by implying that they are weak or overly feminine (Hall, 1997).
Krishna Swamy: “Krishna Swamy” is a respectful way to identify Lord Krishna. He is a major god in Hinduism and is worshipped as the eighth avatar of Vishnu. The term is most common in South India, and is usually referred to in the names of temples where Krishna is the deity being worshipped. For example, the Ambalappuzha Sree Krishna Swamy Temple in Kerala is known for unique rituals and offerings (Ramaswamy, 2013).
Maulana: A Maulana is an honorific title used in South Asia when addressing a respected Muslim scholar or religious leader. Derived from Arabic, maulana means “our master” and implies authority and sincere regard for the sacred act, not to be taken lightly in any religious context (Merriam-Webster, n.d.).
Mohini: Mohini is the only female avatar of the Hindu god Vishnu, portrayed as a shape-shifting enchantress who uses her physical beauty to distract demons, and later, gives the nectar of immortality to the gods. Her most famous show is during the churning of the ocean (Samudra Manthan), where she plays an important role in restoring the balance of the cosmos (Doniger, 1999).
Namaz: Namaz is the Persian name for the Islamic ritual prayer, also known in Arabic as Salah. Namaz refers to the five daily prayers that Muslims perform and are intended to facilitate personal spirituality and discipline (Chittick and Murata, 1994).
Shraddham: Śrāddha or Shraddham is a Hindu ancestral ritual traditionally performed by the male descendants of a household. This practice is primarily found in Brahmin caste families and serves as a sacred act to maintain the spiritual continuity of the family’s lineage and honor the deceased (Holdrege, 1996). The Śrāddha or Shraddham is usually carried out by the eldest son of the Brahmin household, as they are considered to be in charge of spiritual tasks dedicated to ancestors (Knipe, 2015).
Śloka: A śloka is a metrical verse form found in classical Sanskrit literature. A śloka typically consists of 32 syllables arranged into four lines. Ślokas are the most widely used verse form in Sanskrit epic texts, including the Mahabharata and the Ramayana, and a variety of other Hindu scriptures, including scriptures of philosophy (Hopkins, 1901).
Zina: Zina is to have unlawful sexual intercourse, which occurs outside the bounds of marriage, including adultery and fornication. It is considered a major sin in Islam and is regulated through Islamic legal determinations and punishments (Kamali, 2008).
