# Supplementary material for: “Praying in or preying on my skin”: a narrative study of transgender and gender non-conforming individuals’ experiences with religion and gender identity in India
Source: Front Sociol. 2025 Jul 25;10:1642404. doi: 10.3389/fsoc.2025.1642404 (PMC12331479; doi:10.3389/fsoc.2025.1642404)
Supplement: Supplementary file 1 [file Supplementary_file_1.pdf]

Appendix A

Coding Tree

Main Theme 1: God’s Silence

├── Sub-theme 1.1: Unanswered Prayers for Change

│ ├── “Begged Allah to make me a boy”

│ ├── “Prayed to God to fix me”

│ └── “Woke up feeling the same”

├── Sub-theme 1.2: Feeling Abandoned by God

│ ├── “Maybe Allah doesn’t want to hear me”

│ ├── “God did not consider me worthy”

│ └── “God didn’t build me as perfect”

Main Theme 2: Spiritual Exile

├── Sub-theme 2.1: Rejection by Religious Community

│ ├── “Taken to a maulana for spiritual correction”

│ ├── “Christian counsellor told my parents I was brainwashed”

│ └── “Forced to confess my identity was sinful”

├── Sub-theme 2.2: Forced Disidentification from Sacred Roles

│ ├── “Not allowed to light the lamp at Shraddham”

│ ├── “Removed from Christmas prayer at home”

│ └── “Like I’d been spiritually erased”

Main Theme 3: Reclamation of Sacred Identity

├── Sub-theme 3.1: Personal Spiritual Agency

│ ├── “I still pray to Allah in my own way”

│ ├── "Changed the pronouns in hymns"

│ └── "He's still with me—in my mirror, my saree"

├── Sub-theme 3.2: Community and Counter-Theology

│ ├── "Queer Muslim Zoom group"

│ ├── "Queer Hindus doing aarti together"

│ └── "Interfaith queer circles reading Psalms"

Cross-cutting Theoretical Codes

├── Intersectionality

│ ├── Caste- and class-based exclusion

│ └── Rural vs. urban disparities

├── Queer Theology

│ ├── Reinterpretation of scripture

│ └── Sacredness through defiance and re-embodiment

├── Postcolonial Feminism

│ └── Colonial legacies shaping homonationalist religious norms
